# Melatonin Regulates Lipid Metabolism in Porcine Cumulus–Oocyte Complexes via the Melatonin Receptor 2

**DOI:** 10.3390/antiox11040687

**Published:** 2022-03-31

**Authors:** Jun-Xue Jin, Jing-Tao Sun, Chao-Qian Jiang, Hong-Di Cui, Ya Bian, Sanghoon Lee, Lianjin Zhang, Byeong Chun Lee, Zhong-Hua Liu

**Affiliations:** 1 Key Laboratory of Animal Cellular and Genetics Engineering of Heilongjiang Province, College of Life Science, Northeast Agricultural University, Harbin 150030, China; sunjt@neau.edu.cn (J.-T.S.); jiangchaoqianneau@163.com (C.-Q.J.); cuihongdi217@163.com (H.-D.C.); bianya1214@163.com (Y.B.); 2 Department of Theriogenology and Biotechnology, College of Veterinary Medicine, Seoul National University, Seoul 08826, Korea; sanghoon@cnu.ac.kr (S.L.); bclee@snu.ac.kr (B.C.L.); 3 Laboratory of Theriogenology, College of Veterinary Medicine, Chungnam National University, Daejeon 34134, Korea; 4 Department of Chemistry, Korea Advanced Institute of Science and Technology, Daejeon 34141, Korea; lianjin729@kaist.ac.kr

**Keywords:** melatonin receptor, lipid metabolism, reactive oxygen species, cytoplasmic maturation, cumulus–oocyte complexes

## Abstract

Previous studies suggest that the inclusion of melatonin (MTn) in in vitro maturation protocols improves the developmental competence of oocytes by scavenging reactive oxygen species (ROS). However, the molecular mechanisms integrating melatonin receptor (MT)-mediated lipid metabolism and redox signaling during in vitro cumulus–oocyte complex (COC) development still remain unclear. Here, we aimed to elucidate the potential role of MTn receptors in lipid metabolic adjustments during in vitro porcine COC development. We observed that MTn-mediated G_s_α–cAMP/PKA signaling facilitated lipolysis primarily through the MT2 receptor and subsequently increased fatty acid (FA) release by hydrolyzing intracellular triglycerides (TGs) in cumulus cells. Furthermore, *CD36* was a critical FA transporter that transported available FAs from cumulus cells to oocytes and promoted de novo TG synthesis in the latter. In addition, MTn regulated lipogenesis and intracellular lipolysis to maintain lipid homeostasis and limit ROS production, thereby supporting oocyte cytoplasmic maturation and the subsequent embryo development. Taken together, these findings provide insight into the possible mechanism integrating MT2-mediated lipid homeostasis and redox signaling, which limits ROS production during in vitro COC development. Therefore, understanding the dynamics of the interactions between lipid homeostasis and redox signaling driven by MT2 is necessary in order to predict drug targets and the effects of therapeutics used to improve female reproductive health.

## 1. Introduction

Oocyte maturation is an extremely complex process, controlled by numerous molecular factors, which is accompanied by the morphological transformation, redistribution and migration of organelles. Lipid droplets (LDs) are crucial organelles that provide indispensable energy substrates for oocyte development, especially in species with lipid-rich oocytes [1,2]. However, excessive LD content has been correlated with an impaired oocyte developmental competence and low cryosurvival [3]. Compared with oocytes developing in vivo, oocytes via in vitro maturation (IVM) exhibit metabolic abnormalities, such as lipid accumulation-induced lipotoxicity, which lead to developmental arrest [4]. Similar events have been reported during in vivo oocyte maturation, wherein cumulus–oocyte complexes (COCs) exposed to lipid-rich environments, such as the follicular fluid of obese women, exhibited an increased infertility rate [5,6,7]. Therefore, understanding lipid metabolism during oocyte and embryo development in a specific microenvironment is able to facilitate the growth of assisted reproductive technology and promote the development of the livestock industry [8,9].

A porcine oocyte contains 161 ± 18 ng of endogenous lipids, mostly in the triglyceride (TG) form, which is much higher than the lipid content reported in other species [3,10]. Therefore, lipid-rich porcine oocytes serve as an excellent model for the study of the role of lipid and fatty acid (FA) metabolism in mammals [1,3]. During oocyte maturation, the transport of FAs, the cumulus cells, to oocytes is mediated by transzonal projections, fatty acid-binding proteins (FABPs) and/or fatty acid traslocase/CD36 [4,11]. Thereafter, the oocytes synthesize LDs de novo using FAs derived from liquid–liquid phase separation and lipids accumulated in the membrane of the endoplasmic reticulum [3,12]. Consequently, cumulus cells are highly involved in coordinating oocytes and their development [13]. Simultaneously, lipases (mainly through ATGL and HSL) activation in oocytes dramatically reduces the size of LDs through TG hydrolysis, and the released FAs are transported into mitochondria where they undergo β-oxidation and produce ATP [1,14]. Therefore, lipid homeostasis represents a well-orchestrated interaction between lipogenesis and lipolysis, which is extremely critical for oocyte maturation and the subsequent embryo development.

Melatonin (*N*-acetyl-5-methoxytryptamine, MTn) is a natural hormone primarily secreted by the pineal gland [15], which regulates physiological processes, including endocrine regulation, disease resistance and cellular metabolism, through MTn receptors [1,16,17,18,19]. In women, MTn is an efficient indicator of positive fertilization outcomes and is also associated with oocyte quality and the development of healthy embryos [20,21,22,23]. However, until recently, the beneficial effects of MTn on the female reproductive system have been attributed to indirect, antioxidant effects, and thus limited information is available on the mechanisms underlying the direct role of MTn receptors in porcine COC development. Therefore, this study aimed to investigate the regulatory network integrating MTn receptor-mediated lipid metabolism and redox signaling during in vitro COC development.

## 2. Materials and Methods

### 2.1. Chemicals

All chemicals and reagents were obtained from Sigma-Aldrich Chemical Company (St. Louis, MO, USA), unless otherwise stated. Porcine ovaries were obtained from a local slaughterhouse, and no experiments were performed on live animals.

To evaluate the effects of MTn on COC development during IVM, we conducted experiments with six chemical treatments: control (non-treatment), MTn, a MTn receptor antagonist luzindole (Lu, an antagonist for both MT1 and MT2), another MTn receptor antagonist 4P-PDOT (an MT2-specific antagonist), MTn with luzindole (MTn + Lu) and MTn with 4P-PDOT (MTn + 4P).

### 2.2. IVM of Porcine Oocytes

Porcine ovaries were maintained at 30–37 °C during transportation, and small antral follicles (diameter: 3–6 mm) were aspirated using a syringe. COCs, with numerous layers of cumulus cells, were collected; washed using tissue culture medium-199 (TCM-199; Invitrogen, Carlsbad, CA, USA) containing 0.3% polyvinyl alcohol (PVA), 10 mM HEPES and 1% penicillin–streptomycin; and transferred into culture dishes containing IVM medium (TCM-199 containing 10 IU/mL luteinizing hormone, 10 IU/mL follicle stimulating hormone, 10% porcine follicular fluid and 0.91 mM sodium pyruvate). Pooled 50 COCs/condition was then cultured in an incubator at 5% CO_2_, 100% RH and 38.5 °C for 42 h (with or without 10^−^^9^ M MTn or 10^−^^9^ M MTn antagonist).

### 2.3. Assessment of Cumulus Cell Expansion

After 42 h IVM, the cumulus expansion index (CEI) was recorded as CEI grades ranging from 0 to 4. A CEI grade of 0 indicated no cell expansion, characterized by the detachment of cumulus cells from the oocyte, which presumed a flattened monolayer of fibroblast-like morphology, leaving a partially or fully denuded oocyte. A CEI grade of 1 indicated no cell expansion, with spherical cumulus cells closely packed around the oocyte. For CEI grade 2 complexes, only the outermost layers of cumulus cells exhibited expansion, whereas CEI grade 3 complexes exhibited prominent expansion in all cell layers except corona radiata (cells most proximal to the oocyte), and CEI grade 4 complexes indicated the maximum degree of expansion, including cell expansion in corona radiata.

### 2.4. Assessment of Nuclear Maturation

After 42 h IVM, cumulus cells were separated from the oocyte in a pipette using 0.1% hyaluronidase. Subsequently, based on microscopic observations, nuclei were classified into the following categories: immature, metaphase II (MII), or others (degenerated).

### 2.5. Parthenogenetic Activation (PA) and In Vitro Culture (IVC)

Denuded porcine MII oocytes were subjected to electrical activation using a single direct current pulse of 1.5 kV/cm for 60 µs. Subsequently, the activated oocytes were placed into culture dishes containing 500 µL porcine zygote medium 3 per well and incubated at 38.5 °C at 100% RH and 5% CO_2_ for 7 days. Cleavage and blastocyst formation rates were determined on Day 2 and Day 7, respectively. To count the total cell numbers of blastocysts, they were collected on Day 7 and stained with 5 μg/mL Hoechst-33342 for 10 min. Then the blastocysts were mounted on glass slides in a drop of 100% glycerol, compressed gently with a cover slip and observed under a fluorescence microscope (Nikon, Tokyo, Japan) at magnification ×400 to count cell nuclei.

### 2.6. LD, FA and ATP Staining

After 42 h IVM, COCs or denuded oocytes were first fixed using 4% paraformaldehyde (PFA) for 4 h and then transferred to phosphate buffer saline (PBS) supplemented with 10 µg/mL BODIPY-LD (BODIPY 493/503; D3922; Molecular Probes, Eugene, OR, USA), 6 µM BODIPY-FA (BODIPY 558/568 C12; D3835; Molecular Probes), or 500 nM BODIPY-ATP (BODIPY FL ATP; A12410; Molecular Probes) for 1 h. Thereafter, COCs or oocytes were washed and mounted on cover slips, and images were captured using an epifluorescence microscope (TE2000-S; Nikon). The fluorescence intensities were measured using ImageJ software (version 1.46r; National Institutes of Health, Bethesda, MD, USA).

### 2.7. Detection of ROS and GSH Levels in the Oocytes

ROS and GSH levels in matured oocytes were detected using H2DCFDA (Invitrogen) and Cell Tracker Blue CMF2HC (Invitrogen), respectively. Each group was first treated with 10 µM H2DCFDA and 10 µM Cell Tracker Blue for 30 min and then transferred to 4 µL PBS. Images were captured using an epifluorescence microscope. The fluorescence intensities were measured using ImageJ software. The excitation/emission wavelengths were 371/464 nm for CMF2HC and 492–495/517–527 nm for H2DCFDA.

### 2.8. Immunofluorescence (IF) Staining

After 42 h IVM, porcine COCs or oocytes fixed using 4% PFA were placed in 1% Triton X-100 for 30 min, followed by treatment with 2% bovine serum albumin (BSA)–PBS to block non-specific sites and overnight incubation (4 °C) with the primary antibody. After washing thrice with 2% BSA–PBS solution for 5 min, the porcine COCs and oocytes were incubated in primary (MT1, MT2, G_s_α, PKA, ATGL, HSL, PLIN A + B, SREBP1, PGC1α, GDF9 and BMP15) and secondary antibodies (donkey anti-rabbit IgG) for 2 h, respectively. The list of antibodies is presented in Table S1. In addition, oocyte nuclei were labeled with 10 µg/mL Hoechst 33342 for 10 min. Oocytes were washed 3 times in PBS and then mounted on glass slides and evaluated with the same exposure times and adjustments under an epifluorescence microscope (TE2000-S; Nikon). Images were captured using an epifluorescence microscope. The fluorescence intensities were measured using ImageJ software.

### 2.9. Western Blotting (WB)

After 42 h IVM, cumulus cells from 50 COCs for each treatment were washed several times with 1% PVA–PBS and lysed in 60 μL of lysis buffer (20 mM glycerol phosphate, 1 mM EDTA, 150 mM NaCl, 10% glycerol, 20 mM Hepes, 1% Triton X-100 and 2 mM EGTA), containing 0.6 μL of 100 nM phenylmethylsulfonyl fluoride (Beyotime, Haimen, China), for 3 h. The samples were then boiled in water for 5 min at 100 °C. About 1 μg total protein from each sample was loaded onto 12% ExpressPlusTM PAGE Gel (GenScript, Nanjing, China) and transferred to a nitrocellulose (NC) membrane (Millipore, Bedford, MA, USA). Non-specific sites on the NC membrane were blocked using tris-buffer saline plus 0.05% Tween 20 (TBST) and 5% BSA, then incubated overnight with primary antibody (β-ACTIN, MT1, MT2, G_s_α, PKA, ATGL, HSL, PLIN A + B) at 4 °C. The NC membrane was washed thrice using TBST and incubated with horseradish peroxidase-conjugated secondary antibodies. Then they were incubated in SuperSignal West Femto Maximum Sensitivity substrate (Thermo Scientific, Waltham, MA, USA) for 10 min, then scanned using Fujifilm LAS-3000 Imaging System (Fuji, Edison, NJ, USA). The intensity of each specific band was then quantified using Image Gauge version 3.46 software. All experiments were repeated at least three times. The list of antibodies is presented in Table S1.

### 2.10. Real-Time Polymerase Chain Reaction (RT-PCR)

For the analysis of gene expression, the total mRNAs were separately extracted from oocytes and cumulus cells for each group using TRIzol reagent (Invitrogen) according to the manufacturer’s protocol, and the total mRNA concentration was quantified using a NanoDrop 2000 Spectrophotometer (Thermo Fisher Scientific, Wilmington, DE, USA). The following complementary DNA (cDNA) was produced using amfiRivert cDNA Synthesis Platinum Master Mix (GenDEPOT, Barker, TX, USA). A PCR plate (Micro-Amp Optical 96-Well Reaction Plate, Singapore) was made by adding 1 µL cDNA, 0.4 µL (10 pmol/µL) forward primer, 0.4 µL (10 pmol/µL) reverse primer, 10 µL SYBR Premix Ex Taq (TaKaRa, Otsu, Japan) and 8.2 µL Nuclease-free water (NFW; Ambion, Austin, TX, USA), and then amplified on a StepOneTM Real-Time PCR System (Applied Biosystems, Waltham, MA, USA). The amplification protocol included an initial denaturation step for 10 min at 95 °C followed by 40 cycles consisting of denaturation for 15 s at 95 °C, annealing for 1 min at 60 °C and extension for 1 min at 72 °C. The expression of each target gene was quantified relative to the reference gene *GAPDH* (for cumulus cells) or *RN18S* (for oocytes) using the equation, R = 2^−^^ΔΔCt^. For ease of comparison, the average expression level of each gene from the control group was set as 1. Expression values were normalized to those of *GAPDH* or *RN18S*.

### 2.11. Statistical Analyses

Data are represented as mean values ± standard error of the mean (SEM). Significant differences were determined using Tukey’s Honesty Significant Difference (HSD) test following a parametric one-way ANOVA with the statistical software SPSS 17.0 (SPSS Inc., Chicago, IL, USA). The gene expression was compared by Student’s t-test in oocytes. Differences with *p* < 0.05 were considered statistically significant.

## 3. Results

### 3.1. Effects of MTn on Cumulus Cells, Oocytes and Embryo Development

**Figure 1:**
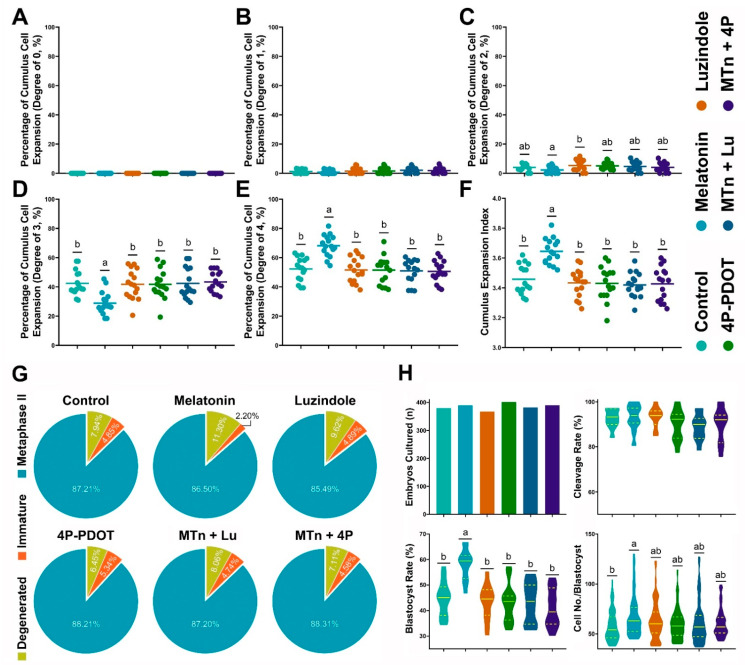
Effects of melatonin on cumulus cells expansion, nuclear maturation and embryo development after parthenogenetic activation. (**A**–**F**) Cumulus expansion index. (**G**) Oocyte nuclear maturation. (**H**) Embryo development. Melatonin/MTn, 10^−^^9^ mol/L melatonin; Luzindole/Lu, 10^−^^9^ mol/L Luzindole; 4P-PDOT/4P, 10^−^^9^ mol/L 4P-PDOT. Different letters denote a significant difference (*p* < 0.05).

The effects of MTn on cumulus cell expansion were evaluated using a total of 3612 COCs in 16 replicates. As shown in Figure 1A–F, MTn treatment significantly increased the proportion of CEI grade 4 COCs (MTn: 68.10 ± 1.83% versus control: 52.24 ± 2.02%, Luzindole: 51.55 ± 2.10%, 4P-PDOT: 51.51 ± 2.34%, MTn + Lu: 50.92 ± 2.01% and MTn + 4P: 50.58 ± 1.83%; *p* < 0.05) and significantly decreased the proportion of CEI grade 3 COCs compared to other treatments (MTn: 28.90 ± 1.92% versus Control: 42.44 ± 2.07%, Luzindole: 41.70 ± 2.53%, 4P-PDOT: 41.71 ± 2.47%, MTn + Lu: 42.35 ± 2.53% and MTn + 4P: 43.45 ± 1.72; *p* < 0.05). Therefore, MTn significantly increased the total CEI compared to other treatments (MTn: 3.64 ± 0.02 versus Control: 3.46 ± 0.02, Luzindole: 3.43 ± 0.02, 4P-PDOT: 3.43 ± 0.03, MTn + Lu: 3.42 ± 0.02 and MTn + 4P: 3.43 ± 0.03; *p* < 0.05).

Nuclear maturation was evaluated after IVM using a total of 3815 COCs in 17 replicates. No significant difference in nuclear maturation was observed among the treatments (Control: 87.2 ± 2.1%, MTn: 86.6 ± 2.2%, Luzindole: 85.3 ± 1.7%, 4P-PDOT: 88.2 ± 1.2%, MTn + Lu: 87.1 ± 1.2% and MTn + 4P: 88.1 ± 1.6%; Figure 1G). We further investigated levels of oocyte developmental indicators (GDF9 and BMP15) and oxidative stress markers (GSH and ROS). Compared with the control, MTn + Lu and MTn + 4P treatments, MTn treatment significantly increased GDF9 and BMP15 expression, as well as the intracellular GSH levels, while significantly reducing the ROS levels in oocytes (*p* < 0.05; Figure S2A–G).

A total of 2308 oocytes in 12 replicates were used to determine the effects of MTn on embryo development after PA (Figure 1H). MTn treatment significantly increased the rate of PA-derived blastocyst formation compared to other treatments (MTn: 58.3 ± 1.7% versus Control: 44.7 ± 2.0%, Luzindole: 43.4 ± 2.0%, 4P-PDOT: 43.1 ± 2.2%, MTn + Lu: 43.2 ± 2.3% and MTn + 4P: 40.9 ± 2.2%; *p* < 0.05). Based on these results, we selected control, MTn, MTn + Lu and MTn + 4P treatments for subsequent experiments.

### 3.2. MTn Upregulates Protein Kinase A (PKA) Signaling Pathway via MT2 in Cumulus Cells

**Figure 2:**
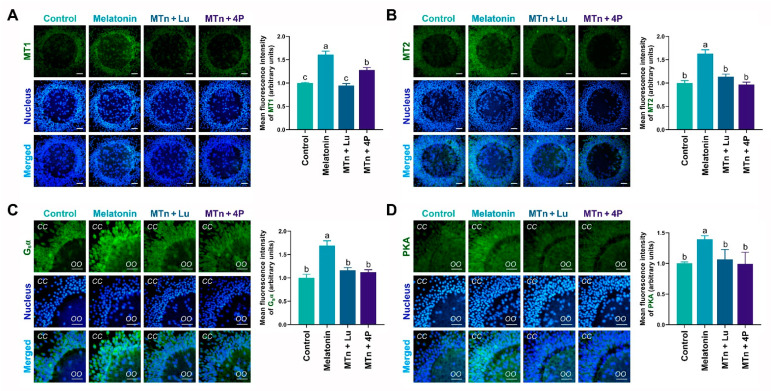
Immunofluorescence analysis of melatonin receptors (**A**,**B**), G_s_α (**C**) and PKA (**D**) proteins in cumulus cells. Melatonin/MTn, 10^−^^9^ mol/L melatonin; Luzindole/Lu, 10^−^^9^ mol/L Luzindole; 4P-PDOT/4P, 10^−^^9^ mol/L 4P-PDOT. Scale bar = 30 μm. Different letters denote significant difference (*p* < 0.05).

The levels of MTn receptors (MT1 and MT2), G-protein stimulatory subunit alpha (G_s_α) and PKA mRNA and protein were detected in cumulus cells using RT-PCR, IF staining and WB. The levels of these proteins significantly increased in cumulus cells following MTn supplementation (*p* < 0.05). By contrast, protein levels were reduced upon treatment with MTn antagonists (Lu and 4P-PDOT) (Figure 2). Furthermore, the results of RT-PCR and WB were similar to those of IF staining (Figure S1). As 4P-PDOT is a selective MT2 antagonist (>300-fold selectivity for MT2 than for the MT1 subtype), the mRNA and protein levels of MT1 decreased in the oocytes exposed to MTn + Lu compared to those exposed to MTn + 4P.

### 3.3. MT2 Promotes Lipolysis in Cumulus Cells

**Figure 3:**
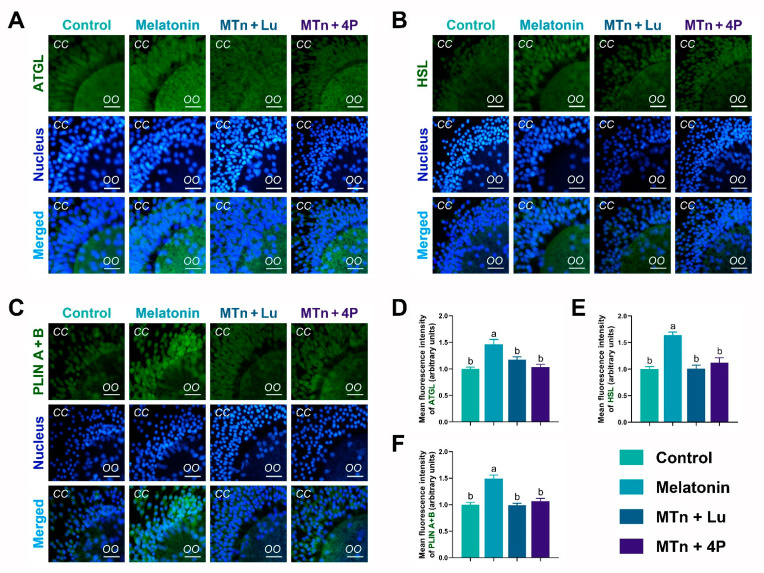
Immunofluorescence analysis of lipolysis proteins in cumulus cells. (**A**,**D**) ATGL, (**B**,**E**) HSL and (**C**,**F**) PLIN A + B. Melatonin/MTn, 10^−^^9^ mol/L melatonin; Luzindole/Lu, 10^−^^9^ mol/L Luzindole; 4P-PDOT/4P, 10^−^^9^ mol/L 4P-PDOT. Scale bar = 30 μm. Different letters denote significant difference (*p* < 0.05).

To estimate lipase activity, the levels of lipolysis-related proteins, including adipose triglyceride lipase (ATGL), hormone-sensitive lipase (HSL) and perilipin (PLIN A + B), were determined in cumulus cells using IF staining. The levels of ATGL, HSL and PLIN A + B were significantly increased upon MTn treatment compared to the control (*p* < 0.05), and this MTn-induced increase was inhibited by MTn antagonists (Figure 3). As shown in Figure S1A, the levels of lipolysis-related proteins obtained using IF staining were the same as those obtained using WB. Moreover, *ATGL* and comparative gene identification-58 (*CGI58*) were upregulated upon MTn treatment compared to other treatments. Although *HSL* was also upregulated upon MTn treatment, there was no significant difference in the expression of *PLIN2*, monoacylglycerol lipase (*MGL*) and lipoprotein lipase (*LPL*) among the treatments (Figure S1B). We also observed that β-oxidation-related genes (*ACADS*, *CPT1B* and *CPT2*) and mitochondrial biogenesis genes (*TFAM*, *PGC1α* and *PRDX2*) were upregulated in cumulus cells following MTn treatment (Figure S1B).

**Figure 4:**
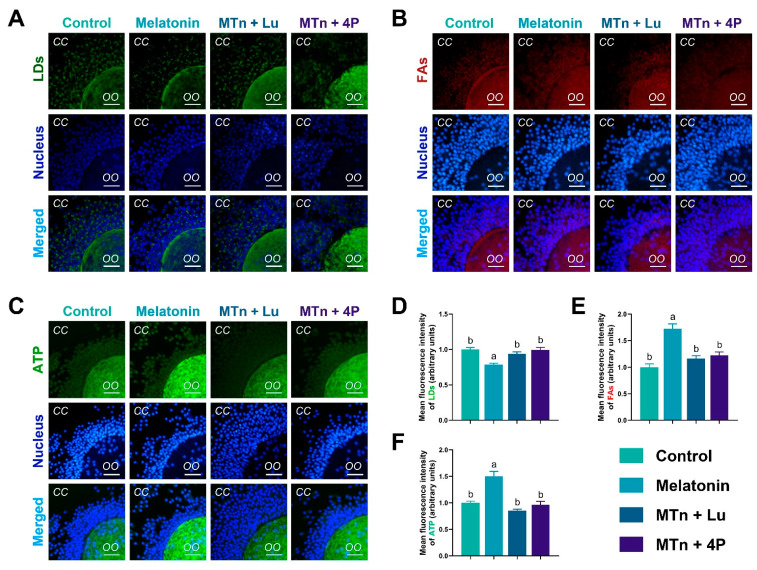
The contents of LDs, FAs and ATP in cumulus cells (**A**–**F**). Melatonin/MTn, 10^−^^9^ mol/L melatonin; Luzindole/Lu, 10^−^^9^ mol/L Luzindole; 4P-PDOT/4P, 10^−^^9^ mol/L 4P-PDOT. Scale bar = 30 μm. Different letters denote significant difference (*p* < 0.05).

To verify lipolysis, we examined the content of LDs, FAs and ATP in cumulus cells. The number of LDs was significantly decreased upon MTn supplementation compared to other treatments (*p* < 0.05) (Figure 4), suggesting that the utilization of LDs was catalyzed by cumulus cell lipases. Additionally, the levels of FAs and ATP increased upon MTn supplementation compared to the control (*p* < 0.05); however, this increase was inhibited by MTn antagonists.

### 3.4. MTn Regulates Lipid Metabolism in Porcine Oocytes

**Figure 5:**
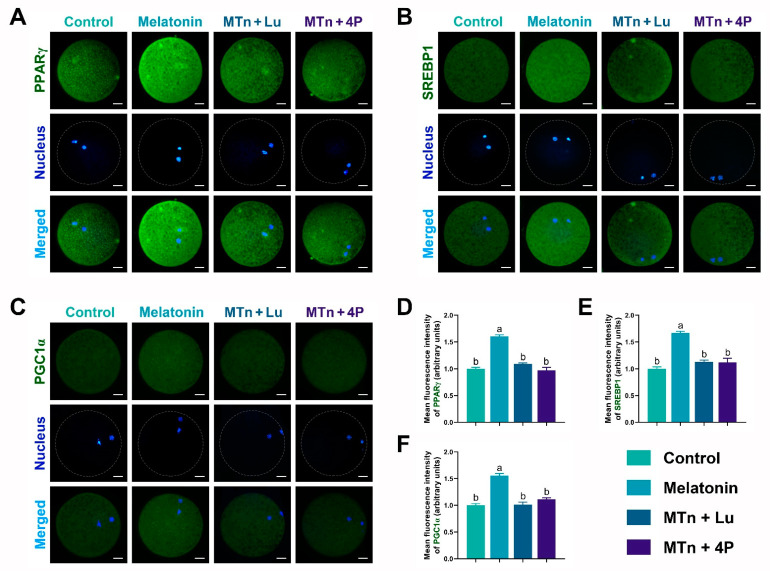
Lipogenesis and mitochondrial biogenesis-related protein expression in oocytes. (**A**,**D**) PPARγ, (**B**,**E**) SREBP1 and (**C**,**F**) PGC1α. Melatonin/MTn, 10^−^^9^ mol/L melatonin; Luzindole/Lu, 10^−^^9^ mol/L Luzindole; 4P-PDOT/4P, 10^−^^9^ mol/L 4P-PDOT. Scale bar = 25 μm. Different letters denote significant difference (*p* < 0.05).

**Figure 6:**
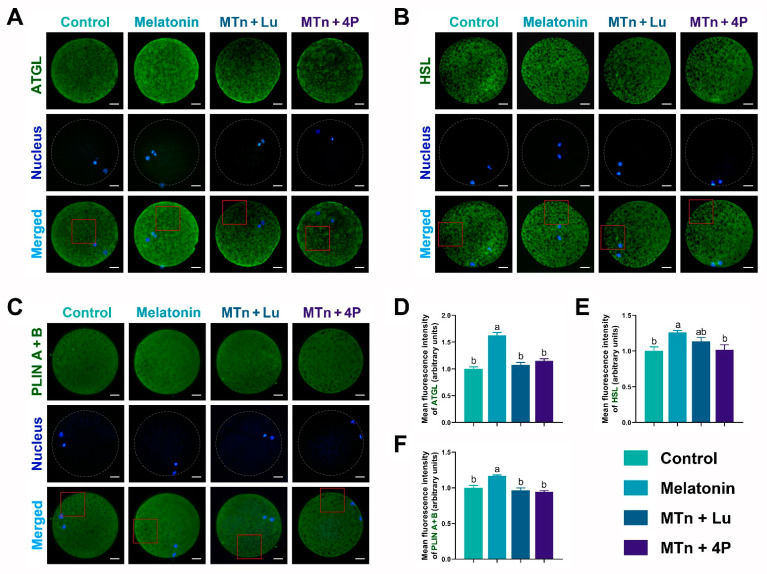
Expression of lipolysis-related proteins in the porcine oocyte. (**A**,**D**) ATGL, (**B**,**E**) HSL and (**C**,**F**) PLIN A + B. Melatonin/MTn, 10^−^^9^ mol/L melatonin; Luzindole/Lu, 10^−^^9^ mol/L Luzindole; 4P-PDOT/4P, 10^−^^9^ mol/L 4P-PDOT. Scale bar = 25 μm. Different letters denote significant difference (*p* < 0.05).

**Figure 7:**
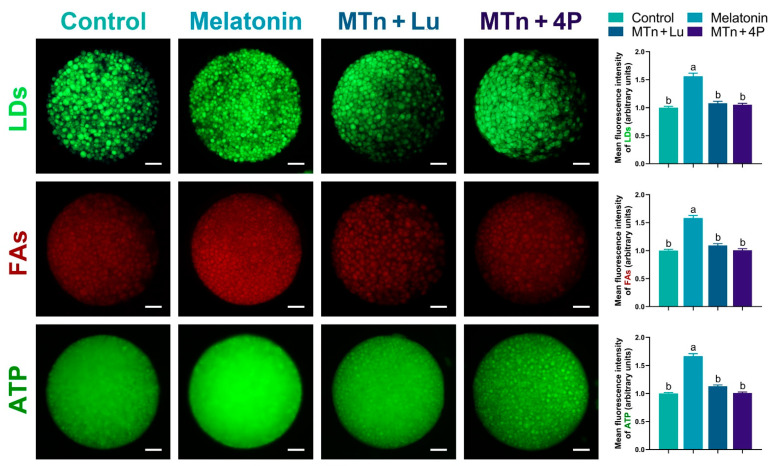
The levels of LDs, FAs and ATP in oocytes. Melatonin/MTn, 10^−^^9^ mol/L melatonin; Luzindole/Lu, 10^−^^9^ mol/L Luzindole; 4P-PDOT/4P, 10^−^^9^ mol/L 4P-PDOT. Scale bar = 25 μm. Different letters denote significant difference (*p* < 0.05).

We performed IF staining in oocytes to determine the levels of PPARγ, SREBP1, ATGL, HSL, PLIN A + B and PGC1α, which were significantly increased upon MTn supplementation (*p* < 0.05); however, the increase was inhibited by MTn antagonists (Figure 5 and Figure 6). Lipases were detected in the ooplasm and localized on the surface of LDs (black dots in red box; Figure 6). Moreover, the content of LDs, FAs and ATP was significantly increased in oocytes upon MTn treatment, and this increase was suppressed by MTn antagonists (Figure 7). Remarkably, although the FA transporter *CD36* was upregulated following MTn treatment compared to the control, this was not the case for *FABP3* and *FABP5* (Figure S2H).

## 4. Discussion

MTn is one of the most powerful antioxidants that reduces oxidative stress by scavenging free radicals. MTn receptors, MT1 and MT2, are G protein-coupled receptors (GPCRs) that receive MTn signals and are involved in a vast array of biological and physiological processes, such as the sleep/wake cycle, hormone secretion, homeostasis and energy balance [24,25,26]. Previously, we demonstrated that MTn promotes lipid metabolism and thereby serves as an essential energy source for oocyte maturation and the subsequent embryonic development [1]. A recent study reported that MTn modulates lipid metabolism in maturing oocytes through the MTn receptors in cumulus cells [9]. Although MTn-mediated lipid metabolism improves COC development, MTn receptor-mediated metabolic pathways and regulatory networks still remain unelucidated. Therefore, in this study, we demonstrated the mechanism underlying MTn receptor-mediated lipid metabolism during in vitro COC development and elucidated its signal transduction pathway.

COC development is an important step for ovum competence that ensures successful insemination and early embryonic development [4,27]. Cumulus cells facilitate a favorable microenvironment necessary for oocyte growth and development by regulating metabolic substrates, eliminating toxic metabolites and modulating environmental effects [28]. Furthermore, cumulus cells are closely associated with the oocytes through gap junctions, which allow a bidirectional paracrine signaling, thereby regulating several processes, such as chromatin remodeling and RNA synthesis, during antral follicle growth [29,30]. A high degree of cumulus cell expansion is a critical biological event during oocyte nuclear and cytoplasmic maturation [31,32]. By contrast, less expansion or the absence of cumulus cells exerts a negative influence on oocyte nuclear maturation, cytoplasmic maturation and early embryonic development after fertilization [33].

We observed that MTn supplementation significantly increased the total CEI by improving the competence of cumulus cell expansion compared to the control. However, the increase was impeded upon the addition of MTn receptor antagonists. Therefore, we hypothesized that the acquisition of high-quality oocytes was closely associated with the complete expansion of cumulus cells after MTn supplementation. Sanchez-Lazo et al. demonstrated the different lipid profiles of cumulus cells before and after IVM; they suggested that lipid metabolism in cumulus cells was critical for homeostasis and influenced meiosis progression in oocytes [34]. Similarly, Auclair et al. verified that the absence of cumulus cells during IVM affected lipogenesis and lipolytic activity in bovine oocytes [30]. Furthermore, a transcriptomic study reported that a number of genes involved in cellular metabolism were differentially expressed between bovine COCs and denuded oocytes after IVM [35]. Therefore, understanding metabolic aberrations in cells closely associated with oocytes provides insight into the natural environment maintained during oocyte maturation [28].

Previously, we elucidated that both MTn receptors *MT1* and *MT2* were expressed in porcine cumulus cells; however, *MT1* was not detected in porcine oocytes [36]. In this study, MT1 and MT2 were detected in porcine cumulus cells using IF and WB analyses, and their expression significantly increased upon MTn supplementation. Additionally, the MTn-mediated increase in *MT2* expression was blocked by Lu and 4P-PDOT, whereas the MTn-mediated *MT1* expression was completely suppressed by Lu and only partially suppressed by 4P-PDOT. Therefore, we further investigated the MTn receptor-mediated lipid metabolic signaling networks in cumulus cells and elucidated the regulatory pathway integrating the lipid profiles of cumulus cells with the developmental regulation of oocytes.

Lipolysis is defined as the enzyme-catalyzed hydrolysis of TGs, which results in the generation of FAs and glycerol by PKA stimulation [37]. Lipolytic signaling is particularly important in several non-adipose tissues [38], wherein adenylate cyclase (AC), which is downstream to GPCRs, is primarily activated upon interaction with the G_s_α [39]. The secondary messenger cyclic adenosine monophosphate (cAMP), generated by AC activation, regulates several effectors, the most studied of which is cAMP-dependent PKA [40]. PKA regulates numerous pathological and physiological processes in mammals and homeostasis in eukaryotes [41,42]. Following PKA stimulation, HSL is translocated from the ooplasm to the LD surface, where it interacts with the members of the PLIN family to stimulate lipolysis [43,44]. Moreover, after PKA activation, ATGL indirectly interacts with PLIN through the co-factor CGI58 in the adipocytes [45]. Therefore, the lipolysis of LDs in COCs is catalyzed by lipolytic enzymes, including HSL and ATGL [46]. In this study, our results indicated that MTn significantly increased G_s_α levels in cumulus cells, which upregulated the cAMP/PKA pathway, and then activated lipolytic processes by activating lipases (ATGL and HSL) and their co-factors (PLIN and CGI58). Consequently, MTn significantly decreased the number of intracellular LDs, increased FA and ATP levels and enhanced the expression of genes (*MT1*, *MT2*, *G_s_α*, *PRKAR1A*, *PRKAR1B*, *PRKACA*, *ATGL*, *HSL*, *CPT1B*, *CPT2*, *ACADS*, *PGC1α*, *TFAM* and *PRDX2*) involved in β-oxidation in cumulus cells. However, these MTn-mediated effects were completely suppressed by Lu and 4P-PDOT.

4P-PDOT is an MT2-specific antagonist, whereas Lu is an antagonist for both MT1 and MT2. Therefore, an MTn-mediated G_s_α–PKA signaling pathway facilitated lipolytic processes primarily via MT2 stimulation, producing metabolic substrates as an essential energy source for cumulus cell development during IVM. Similar to these findings, He et al. demonstrated that the functions of porcine granulosa cells were primarily regulated by MT2 [47], suggesting that MT2 probably mediated porcine cumulus cell expansion upon MTn treatment. In addition, we previously elucidated that cumulus cell expansion and embryonic development were promoted by MTn treatment via the activation of a sonic hedgehog signaling pathway [36]. We also reported that MT2 mediated the stimulatory effects of MTn on porcine cumulus cell expansion and the subsequent embryo development [48]. In this study, we demonstrated that MTn improved oocyte cytoplasmic maturation by increasing GDF9 and BMP15 levels. These findings suggest that lipid metabolism in cumulus cells might strongly regulate oocyte development through MT2.

Cumulus cells balance FA accumulation in oocytes using special “channels” such as gap junctions and transzonal projections [4]. In addition, the intracellular lipids in the oocytes release FAs by the activation of lipolysis, which provides the energy for oocyte maturation and development [1,3,49,50]. A previous study revealed that disrupting gap junctions using carbenoxolone reduced LD content and compromised developmental competence in porcine oocytes [9]. Furthermore, in bovine oocytes, the disruption of transzonal projections decreased lipid accumulation during IVM [4]. Although these studies suggest that lipid accumulation in oocytes primarily contributed to the FA release by cumulus cells, the mechanisms underlying the transport of FAs from cumulus cells to the ooplasm remain unknown.

Recent studies have shown that FA binding proteins are characterized by intra- and extra-cellular FA transport. In bovine COCs, FABP3, not FABP5, was localized in the transzonal projections, where it altered FA traffic, resulting in lipid accumulation in the oocytes [4]. In addition, CD36, a fatty acid translocase located in the cell membranes of oocytes, facilitates the uptake of long chain FAs [38] by the oocytes [51]. In the present study, the expression of *CD36*, not *FABP3* or *FABP5*, was significantly increased upon MTn treatment. Thus, CD36 might be an important FA transporter between cumulus cells and oocytes in pigs. Moreover, we detected several lipogenetic (PPARγ and SREBP1) and lipolytic proteins (ATGL, HSL and PLIN A + B) in the oocytes, the levels of which were significantly increased upon MTn treatment. This led to increased FA and ATP generation, thereby improving the oocyte quality and blastocyst formation rate.

According to Jiang et al., MTn regulates redox homeostasis by improving mitochondrial function and reducing the damage resulting from ROS generation in human oocytes [52]. Moreover, PGC1α activates Nrf2 for the precursors of mitochondrial translation factor A (TFAM) to activate mitochondrial function, maintenance and biogenesis [53,54]. Previously, we reported that MTn also regulated peroxisomal activities by stimulating NRF2 signaling, which reduced oxidative stress in porcine COCs [55]. Similar to these findings, in this study, MTn supplementation increased levels of PGC1α, the expression of *PGC1α* and *TFAM*, and intracellular GSH levels; it also decreased the level of ROS. Taken together, the findings of this study indicate that MTn plays a role in balancing lipogenesis and intracellular lipolysis in order to maintain lipid homeostasis and limit ROS production, thereby supporting oocyte cytoplasmic maturation and the subsequent embryo development.

## 5. Conclusions

The present study elucidated the interaction between MT2-mediated lipid homeostasis and redox signaling, which limited ROS production during in vitro COC development. Therefore, understanding the molecular mechanisms underlying the dynamics of the interactions between these metabolic networks driven by MT2 is necessary to predict drug targets and the effects of therapeutics used to improve female reproductive health.

## Data Availability

The data are contained within the article and the supplementary materials.

## Supplementary Materials

The following supporting information can be downloaded at: https://www.mdpi.com/article/10.3390/antiox11040687/s1, Figure S1: Expression of mRNA and proteins in cumulus cells by Real-time PCR and Western blot analysis, respectively, Figure S2: Expression of oocyte developmental indicators and lipid metabolism-related genes in oocytes, Table S1: Information of antibodies for immunofluorescence or western blot.
