# Supplementary material for: Melatonin Regulates Lipid Metabolism in Porcine Cumulus–Oocyte Complexes via the Melatonin Receptor 2
Source: Antioxidants (Basel). 2022 Mar 31;11(4):687. doi: 10.3390/antiox11040687 (PMC9027243; doi:10.3390/antiox11040687)
Supplement: Supplementary file 1 [file antioxidants-11-00687-s001.zip › antioxidants-1596069-supplementary.pdf]

Table S1. Information of antibodies for immunofluorescence or western blot.

**Antibodies**	**Source**	**Catalog No.**
Anti-β-Actin Antibody	Sigma	A1978
MTNR1A (MT1) Polyclonal Antibody	Invitrogen	PA5-77490
MTNR1B (MT2) Polyclonal Antibody	Invitrogen	PA5-77491
Anti-GNAS ($G_s\alpha$)	Abcam	ab235956
Anti-PKA Alpha/Beta/Gamma	Abcam	ab75991
Anti-Adipose Triglyceride Lipase (ATGL)	Abcam	ab207799
Anti-Hormone Sensitive Lipase (HSL)	Abcam	ab45422
Perilipin A/B Polyclonal Antibody	Invitrogen	PA1-1052
Anti-PPAR Gamma Antibody	Abcam	ab209350
SREBP1 Antibody	Novus Biologicals	NB100-2215
Anti-PGC1 Alpha	Abcam	ab54481
Anti-GDF9	Abcam	ab93892
BMP-15 Polyclonal Antibody	Invitrogen	PA5-34401
Donkey anti-Rabbit IgG (H+L) Highly Cross-Adsorbed Secondary Antibody, Alexa Fluor 488	Invitrogen	A-21206
Anti-Mouse IgG-Peroxidase antibody produced in rabbit	Sigma	A9044
Anti-Rabbit IgG-Peroxidase antibody produced in goat	Sigma	A9169

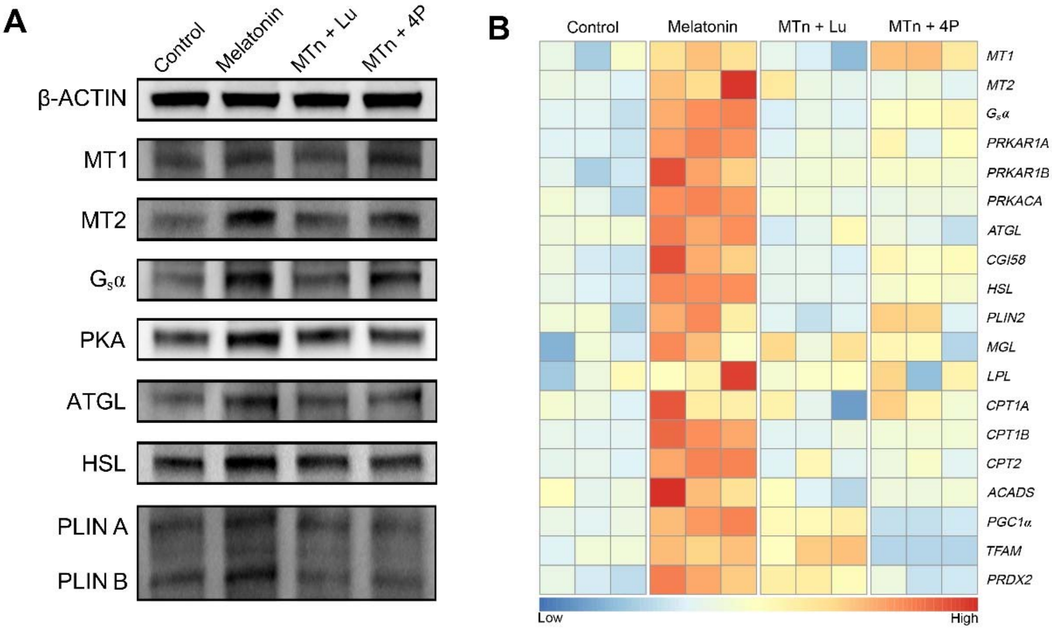

Figure S1. Expression of mRNA and proteins in cumulus cells by Real-time PCR and Western blot analysis, respectively. (A) Relative protein expression and (B) mRNA expression. Melatonin/MTn, 10^{-9} mol/L melatonin; Luzindole/Lu, 10^{-9} mol/L Luzindole; 4P-PDOT/4P, 10^{-9} mol/L 4P-PDOT.

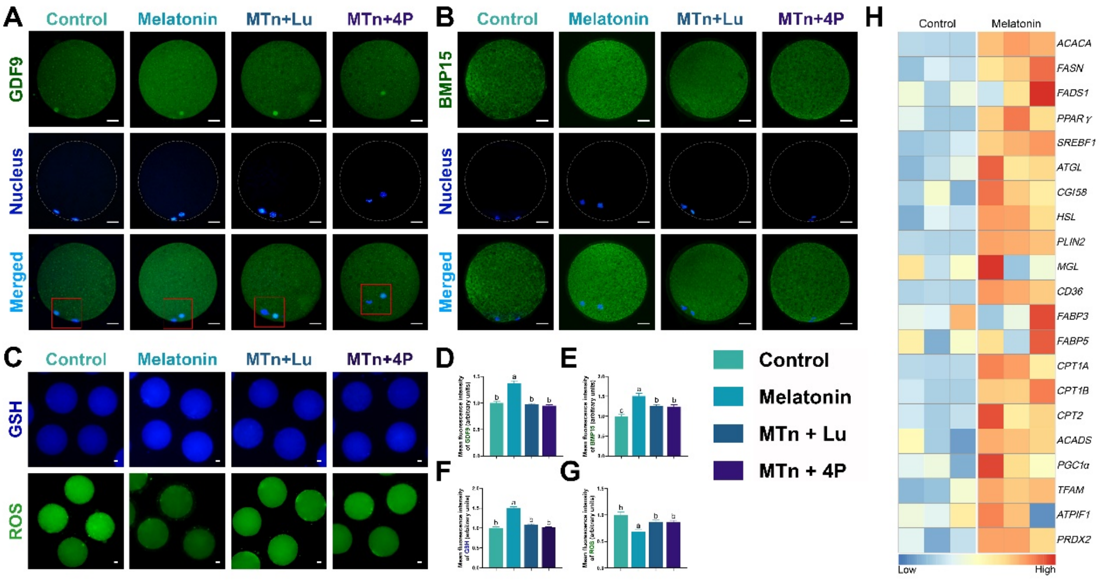

Figure S2. Expression of oocyte developmental indicators and lipid metabolism-related genes in oocytes. (A and D) GDF9, (B and E) BMP15, (C, F and G) GSH and ROS,

and (H) mRNA expression related to lipid metabolism. Melatonin/MTn, 10^{-9} mol/L melatonin; Luzindole/Lu, 10^{-9} mol/L Luzindole; 4P-PDOT/4P, 10^{-9} mol/L 4P-PDOT. Scale bar = 25 μm. Different letters denote significant difference ($P < 0.05$).
